# A novel QTL associated with tolerance to cold-induced seed cracking in the soybean cultivar Toyomizuki

**DOI:** 10.1270/jsbbs.22066

**Published:** 2023-04-25

**Authors:** Naoya Yamaguchi, Yumi Sato, Fumio Taguchi-Shiobara, Kazuki Yamashita, Michio Kawasaki, Masao Ishimoto, Mineo Senda

**Affiliations:** 1 Hokkaido Research Organization Tokachi Agricultural Experiment Station, Shinsei, Memuro-cho, Kasai-gun, Hokkaido 082-0081, Japan; 2 Faculty of Agriculture and Life Science, Hirosaki University, Bunkyo, Hirosaki, Aomori 036-8561, Japan; 3 Institute of Crop Science, The National Agriculture and Food Research Organization, Tsukuba, Ibaraki 305-8518, Japan

**Keywords:** soybean, seed cracking, abiotic stress, tolerance, quantitative trait locus

## Abstract

Low temperatures after flowering cause seed cracking (SC) in soybean. Previously, we reported that proanthocyanidin accumulation on the dorsal side of the seed coat, controlled by the *I* locus, may lead to cracked seeds; and that homozygous *IcIc* alleles at the *I* locus confer SC tolerance in the line Toiku 248. To discover new genes related to SC tolerance, we evaluated the physical and genetic mechanisms of SC tolerance in the cultivar Toyomizuki (genotype *II*). Histological and texture analyses of the seed coat revealed that the ability to maintain hardness and flexibility under low temperature, regardless of proanthocyanidin accumulation in the dorsal seed coat, contributes to SC tolerance in Toyomizuki. This indicated that the SC tolerance mechanism differed between Toyomizuki and Toiku 248. A quantitative trait loci (QTL) analysis of recombinant inbred lines revealed a new, stable QTL related to SC tolerance. The relationship between this new QTL, designated as *qCS8-2*, and SC tolerance was confirmed in residual heterozygous lines. The distance between *qCS8-2* and the previously identified QTL *qCS8-1*, which is likely the *Ic* allele, was estimated to be 2–3 Mb, so it will be possible to pyramid these regions to develop new cultivars with increased SC tolerance.

## Introduction

In soybean (*Glycine max*), low temperatures after flowering result in lower seed quality. For example, the seed coat occasionally becomes pigmented around the hilum under low temperature (Morrison *et al.* 1998, Srinivasan and Arihara 1994, Takahashi 1997, Takahashi and Asanuma 1996). We called this type of seed deterioration “cold-induced seed coat discoloration”, abbreviated as CD (Kasai *et al.* 2009). Seeds affected by CD often show cracking of the pigmented seed coat, and this further reduces seed quality. Cold-induced seed cracking, abbreviated as SC, is another type of seed deterioration (Senda *et al.* 2018, Yamaguchi *et al.* 2014). In typical SC-affected seeds exhibiting the CD phenotype, the seed coat splits in a straight line on the dorsal side, so the two cotyledons are exposed and frequently separated (Senda *et al.* 2018, Yamaguchi *et al.* 2014, 2021). Because the quality of SC-affected seeds is lower than that of CD-affected seeds, they have no commercial value and are discarded, leading to reductions in soybean yield. Therefore, in cold areas where SC occurs, for example, in the Okhotsk area of north-eastern Hokkaido in Japan, cultivars with improved SC tolerance are required for stable seed production.

The inhibitor (*I*) locus controls the distribution of anthocyanins and proanthocyanidins (PAs) in the epidermal layer of the seed coat (Palmer *et al.* 2004, Todd and Vodkin 1993). At the *I* locus, four alleles are known: *I*, *i^i^*, *i^k^* and *i* (Bernard and Weiss 1973). The *I* allele inhibits pigmentation of the hilum and entire seed coat. The *i^i^* and *i^k^* alleles limit pigmentation to the hilum and the saddle-shaped region, respectively, while the *i* allele permits pigmentation of the hilum and entire seed coat. The genotypes of yellow soybean cultivars are *II* (yellow-hilum cultivars) and *i^i^i^i^* (pigmented-hilum cultivars). In yellow soybeans, seed coat pigmentation is inhibited by naturally occurring RNA silencing of chalcone synthase (*CHS*) genes, which is known as CHS silencing (Senda *et al.* 2004, Tuteja *et al.* 2004). A candidate region for the *I* allele was sequenced and designated as *GmIRCHS* (*Glycine max* inverted repeat of *CHS* pseudogene). This sequence was predicted to trigger CHS silencing (Kasai *et al.* 2007, Kurauchi *et al.* 2011). It is likely that CD occurs due to suppression of CHS silencing by low temperature (Kasai *et al.* 2009). Interestingly, a CD-tolerant yellow-hilum cultivar, Toyoharuka, possesses a polymorphic *GmIRCHS* structure (Kasai *et al.* 2009), suggesting that this cultivar has a novel allele at the *I* locus, designated as *Ic* (Yamaguchi *et al.* 2015). The *Ic* allele inhibits pigmentation of the hilum and entire seed coat, and contributes to CD tolerance (Ohnishi *et al.* 2011, Yamaguchi *et al.* 2019).

In our prior study, a phytotron-based assay for evaluation of SC tolerance was developed, in which soybean plants were subjected to a 21-day low-temperature treatment at 10 days after first flower opening (Yamaguchi *et al.* 2014). Using this assay, the SC occurrence mechanism was investigated in the SC-sensitive cultivar Yukihomare (YH) with the *II* genotype (Senda *et al.* 2018). The results showed that the PA accumulation expands to the dorsal side of the seed coat, then subsequent lignin deposition alters the physical properties of the seed coat. These changes result in the straight-line split along the dorsal side of the seed coat at the full-sized seed stage, in other words, the R6 stage (Ritchie *et al.* 1985). After the seed coat splits on the dorsal side, the two cotyledons inside the seed are exposed and then separate during seed desiccation and maturation. The SC-tolerance mechanism was also investigated in the breeding line Toiku 248 (T248) (Senda *et al.* 2018). Because the SC-tolerant line T248 possesses the *IcIc* genotype, PA accumulation and subsequent lignin deposition in the entire seed coat is suppressed under a 21-day low-temperature treatment. Thus, the *Ic* allele may confer SC tolerance as well as CD tolerance (Senda *et al.* 2018). Supporting this possibility, two quantitative trait loci (QTLs) associated with SC tolerance were detected in recombinant inbred lines (RILs) derived from a cross between T248 and YH. One of those QTLs, *qCS8-1*, was mapped near the *I* locus, suggesting that the *Ic* allele may be *qCS8-1* (Yamaguchi *et al.* 2015). However, a single *Ic* allele is insufficient for developing cultivars with improved SC tolerance, so it is important to discover new genes related to SC tolerance that can be pyramided in breeding programs (Yamaguchi *et al.* 2021).

**Fig. 1.**
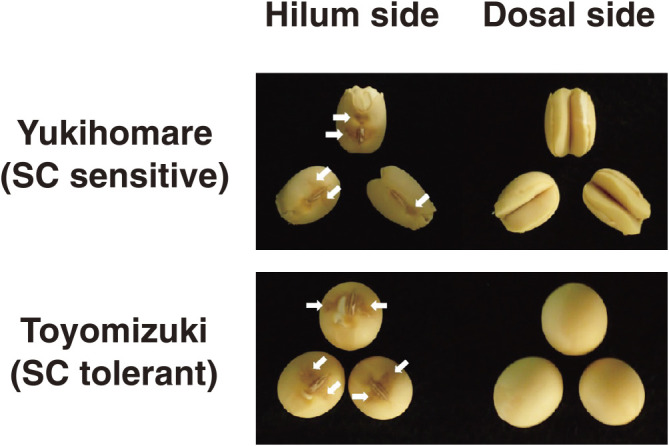
Representative seeds of Yukihomare (YH) and Toyomizuki (TZ) under a 21-day low-temperature treatment. In typical SC-affected seeds of YH, the seed coats are severely split on the dorsal side, and the cotyledons are exposed and separated. White arrow indicates seed coat discoloration around the hilum.

The new SC-tolerant cultivar Toyomizuki (TZ) released in 2012 is a potential source of a novel SC tolerance-related gene other than the *Ic* allele. The TZ cultivar has the *II* genotype at the *I* locus, and exhibits the CD phenotype in the phytotron-based assay, similar to the SC-sensitive YH with the *II* genotype (Fig. 1). In contrast, the SC-tolerant T248 with the *IcIc* genotype does not exhibit CD in the phytotron-based assay (Senda *et al.* 2018, Yamaguchi *et al.* 2014). Thus, the SC tolerance-related gene of TZ is likely to differ from that of T248. In this study, we analyzed the seed coat of TZ under low temperature to determine whether its SC-tolerance mechanism differs from that of T248. Then, using a RIL population derived from a YH and TZ cross, we identified a novel QTL associated with the SC tolerance of TZ. In soybean breeding programs, the use of a DNA marker closely linked to this QTL may allow for the development of new soybean cultivars with improved SC tolerance.

## Materials and Methods

### Plant materials

**Fig. 2.**
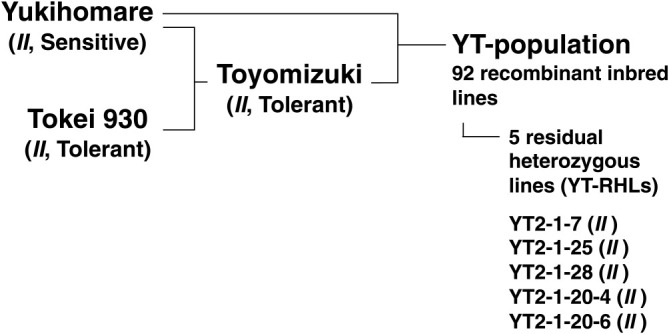
Pedigree of soybean cultivars and lines. Toyomizuki (TZ) is closely related to Yukihomare (YH). All cultivars and lines used in this study had the *II* genotype at the *I* locus, which is shown in parentheses. In TZ, YH and Tokei 930, sensitivity/tolerance to SC is indicated in parentheses.

The parent cultivars YH and TZ were bred at the Tokachi Agricultural Experiment Station, Memuro, Hokkaido, Japan, and were released in 2001 and 2012, respectively (Tanaka *et al.* 2003). The YH and TZ cultivars were respectively registered as nos. 23959 and 27954 at the Hokkaido Research Organization, Japan. A RIL population consisting of 92 lines was bred using the single-seed descent method from a YH × TZ cross, and was designated as the YT-population (Fig. 2), where YT is derived from the initial capital letters of both of the parent cultivars. The generations of the YT-population were F_4_ in 2018 and F_5_ in 2019. The TZ cultivar was bred from a cross between YH and Tokei 930. Both Tokei 930 and TZ are SC tolerant, while YH is SC sensitive. All cultivars and lines used in this study had the *II* genotype at the *I* locus, as confirmed by DNA marker analysis (Ohnishi *et al.* 2011). Neither YH nor TZ has T248 in its pedigree (Fig. 2).

Residual heterozygous lines (RHLs) are bred from a RIL in which the genomic region of interest is segregated while the other regions are fixed (Ikeda *et al.* 2009, Tuinstra *et al.* 1997, Yamanaka *et al.* 2005). In this study, five RHLs were bred from a RIL named YT2, which was heterozygous at the nearest markers to two QTLs, *qCS1-1* and *qCS8-2*. To breed RHLs, DNA was isolated from a leaf of each F_4_ plant (YT2-1) or F_5_ plant (YT2-1-20). The plants were genotyped at Satt179 for *qCS1-1*, and at Satt424 and AW132402 for *qCS8-2*. The F_5_ or F_6_ progeny with homozygous genotypes at *qCS1-1* and *qCS8-2* were designated as YT-RHLs (Fig. 2). The generations of YT2-1-7, YT2-1-25, and YT2-1-28 were F_5_ in 2019. The generations of YT2-1-20-4 and YT2-1-20-6 were F_6_ in 2019.

### Histochemical analysis

For seed coat observation, tissue sections were prepared according to the methods of Senda *et al.* (2017). The procedure for the low-temperature treatment was as follows: 10 days after the flowering date, the potted soybean plants were transferred to a phytotron and grown for 21 days under low-temperature conditions [18°C day (08:00–18:00) and 13°C night (18:00–08:00)], with 55% shading. After the low-temperature treatment, the pots were returned to the following normal conditions: 25°C day (08:00–18:00) and 20°C night (18:00–08:00). Nineteen days later, developing seeds at the R6 stage (500–1000 mg fresh weight) were collected from the pods of YH and TZ. The R6 stage is the reproductive stage of soybean plant development in which seeds are green and full-sized (Ritchie *et al.* 1985). Seed coats of non-dried seeds were directly prepared for embedding in 5% (w/v) agar. After embedding, 50-μm sections were cut with a vibrating microtome and placed on glass slides. Histochemical staining of PAs with 4-dimethylaminocinnamaldehyde (DMACA), which stains PAs blue, was performed as described previously (Senda *et al.* 2017). The stained sections were observed under a microscope (BX51, Olympus, Tokyo, Japan).

### Seed coat penetration test

Penetration testing of seed coats was performed as described previously (Senda *et al.* 2017, 2018). The plants were subjected to the low-temperature treatment as described above, and then returned to the following normal conditions: 25°C day (08:00–18:00) and 20°C night (18:00–08:00). The normal-temperature treatment consisted of 25°C days (08:00–18:00) and 20°C nights (18:00–08:00). Nineteen days after the end of the 21-day low-temperature treatment or 12 days after the end of the 21-day normal-temperature treatment, developing seeds at the R6 stage were removed from the pods of YH and TZ and the seed coats were collected. A texture analyzer (TA.XT *Plus*, Stable Micro Systems, Surrey, UK) was used to measure the resistance of the seed coat to penetration. A P/2N needle probe was pushed through the seed coat from the inner to the outer sides, and the breaking load (g), breaking elongation (mm), and rupture time (s) required for penetration were measured. The Tukey–Kramer test (*P* < 0.05) was used for determination of significant differences among sample groups.

### Evaluation of SC tolerance in the RIL population

The SC tolerance of 92 and 91 RILs was evaluated in 2018 and 2019, respectively. The SC tolerance test was conducted according to Yamaguchi *et al.* (2014). Six seeds per pot were sown in 1/5000a Wagner pots (200 cm^2^ surface area, Fujiwara Scientific Co., Tokyo, Japan) on 28 May in 2018 and 21 May in 2019. Four pots were prepared for each parent cultivar, and one pot was prepared for each RIL. Two weeks after seedling emergence, two plants per pot were selected and were grown in an experimental facility under a plastic roof without walls. At 10 days after the flowering date, the pots were transferred to a phytotron and grown for 21 days under the following low-temperature conditions: 18°C day (08:00–18:00) and 13°C night (18:00–08:00), with 55% shading. After the low-temperature treatment, the pots were returned to the experimental facility, and the plants were grown to maturity. Seeds were harvested from each pot, and the cracked seed rate was calculated as follows: cracked seed rate (%) = (number of cracked seeds/total number of seeds) × 100. For statistical analysis, the cracked seed index (CSI) was calculated by arcsine transformation of the cracked seed rate (Yamaguchi *et al.* 2015). Student’s t-test was used to determine significant differences between the marker genotypes in the RILs.

### Evaluation of SC tolerance in the RHLs

Ten seeds per pot were sown in 25-L plastic pots. Three pots were prepared for each parent cultivar, and two pots were prepared for each RHL. Two weeks after seedling emergence, three plants were selected. Each individual plant was considered as a replicate. The JMP 10 statistical package (SAS Institute 2012) was used for statistical analysis. One-way analysis of variance (ANOVA) was used to test differences in CSI among cultivars and RHLs, or marker genotypes. Significant differences among materials were determined with Tukey’s HSD test (*P* < 0.05).

### Molecular marker analysis and QTL analysis for CSI

DNA extraction and PCR analyses of simple sequence repeat (SSR) markers were performed as described previously (Hwang *et al.* 2009, Sayama *et al.* 2010). Total genomic DNA was isolated from a leaf of each F_3_ RIL using a BioSprint 96 DNA Plant Kit (Qiagen, Hilden, Germany). Polymorphisms of the DNA markers were analyzed using the SSR genotyping panel system (Sayama *et al.* 2011). The F_4_ seeds were harvested from the genotyped F_3_ individuals. A QTL analysis for CSI was performed using the single-marker analysis method in QTL Cartographer version 2.5 (Wang *et al.* 2007). The 59 polymorphic SSR markers were used for these analyses.

## Results

### Histochemical analysis

**Fig. 3.**
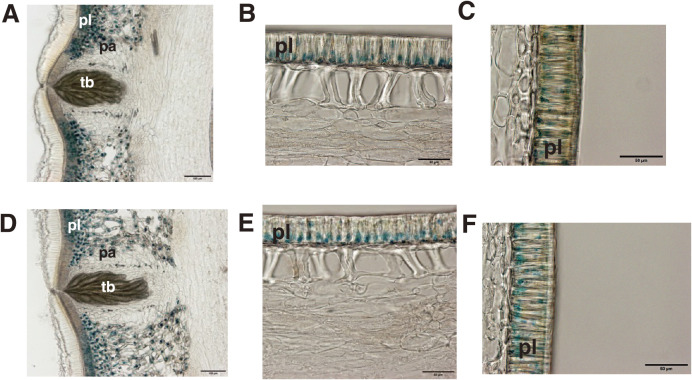
Cross-sections of seed coats of Yukihomare (YH) and Toyomizuki (TZ) grown under low-temperature conditions (YH-L and TZ-L, respectively). Seed coat sections were stained with 4-dimethylaminocinnamaldehyde, which dyes proanthocyanidins blue. (A) YH-L (hilum region), (B) YH-L (middle region), (C) YH-L (dorsal region), (D) TZ-L (hilum region), (E) TZ-L (middle region), (F) TZ-L (dorsal region). pa, parenchyma. pl, palisade layer. tb, tracheid bar. Scale bars are 100 μm (A, D) and 50 μm (B, C, E, F).

In the seeds of YH and TZ without low-temperature treatment, PAs barely accumulated in the seed coat (Supplemental Fig. 1). After the 21-day low-temperature treatment in the phytotron-based assay, PA accumulation in the TZ seeds expanded to the dorsal side of the seed coat, similar to the pattern of PA accumulation in the SC-sensitive cultivar YH (Fig. 3).

### Seed coat penetration test

**Fig. 4.**
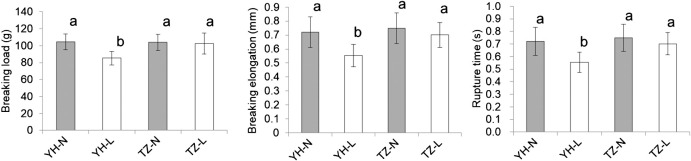
Comparison of physical properties of seed coats. Seed coats from R6-stage seeds of Yukihomare (YH) and Toyomizuki (TZ) grown under normal-temperature conditions (YH-N and TZ-N, respectively), and under low-temperature conditions (YH-L and TZ-L, respectively) were analyzed. Error bars indicate standard deviation (*n* = 62 for YH-N, *n* = 54 for YH-L, *n* = 63 for TZ-N, and *n* = 59 for TZ-L). Different letters indicate significant differences at *P* < 0.05 (Tukey–Kramer test).

To evaluate the physical properties of the seed coat, penetration tests were carried out using a texture analyzer. The breaking load, breaking elongation, and rupture time required for penetration were measured. Breaking load is an index of hardness, and breaking elongation and rupture time are indicators of flexibility (Senda *et al.* 2017, 2018). The breaking load, breaking elongation, and rupture time were significantly lower in the low-temperature treatment than in the normal-temperature treatment in SC-sensitive YH, while these values did not differ significantly between the low- and normal-temperature treatments in SC-tolerant TZ (Fig. 4).

### QTL analysis for CSI in the RIL population

We selected and analyzed 214 SSR markers. Among them, 59 markers were polymorphic between the parents (YH and TZ); the polymorphism rate was 27.6%. The parental cultivars of TZ are YH and Tokei 930 (Fig. 2), indicating that TZ is very closely related to YH. Therefore, a QTL analysis for CSI was performed using the single-marker analysis method.

**Fig. 5.**
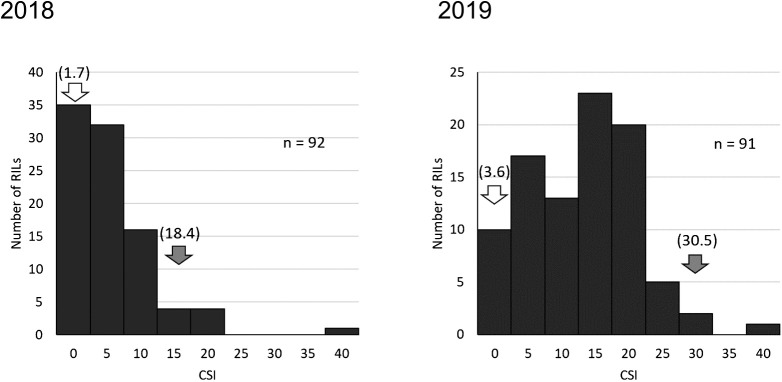
Frequency distribution of cracked seed index (CSI) in each year. Gray and white arrows indicate the CSIs of Yukihomare (YH) and Toyomizuki (TZ), respectively. CSIs of parental cultivars are shown in parentheses.

**Fig. 6.**
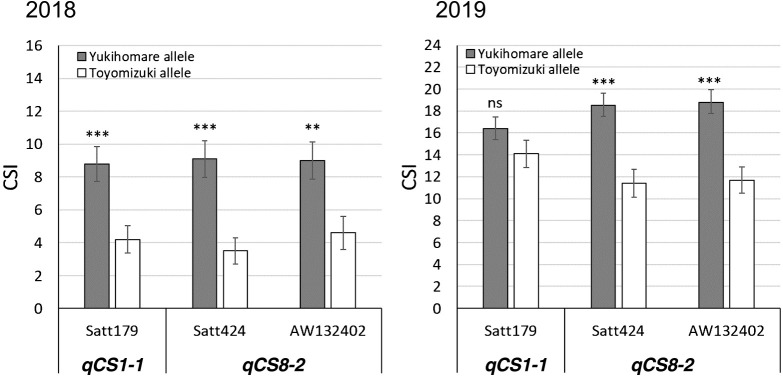
Relationship between *qCS* genotype and cracked-seed index (CSI) in recombinant inbred lines. Satt179 is the nearest marker to *qCS1-1*, and Satt424 and AW132402 are the nearest markers to *qCS8-2*. Error bars indicate standard error. ns, not significant. ** Significant at *P* < 0.01. *** Significant at *P* < 0.001.

Representative seeds of YH (SC sensitive) and TZ (SC tolerant) under low temperature are shown in Fig. 1. The low-temperature treatment caused the seed coats to discolor around the hilum in YH and TZ. The frequency distribution of CSI in each year is shown in Fig. 5. In 2018, the average CSIs for YH and TZ were 18.4 and 1.7, respectively. In 2019, the average CSIs for YH and TZ were 30.5 and 3.6, respectively. The results of the QTL analysis of CSI using 59 SSR markers are shown in Supplemental Table 1. There were six and two significant markers in 2018 and 2019, respectively (Table 1). In 2018, two QTLs were detected on chromosomes Gm01 and Gm08 (Schmutz *et al.* 2010). In 2019, a QTL was detected on Gm08. The QTL on Gm08 was detected at a similar position in both years. We named the QTL on Gm01 *qCS1-1* (Table 1). In our earlier study on a different population, we designated a QTL on Gm08 as *qCS8-1* (Yamaguchi *et al.* 2015). Thus, the QTL detected on Gm08 in this study was designated as *qCS8-2* (Table 1). The nearest marker, Satt179, was used for genotyping *qCS1-1*. The two nearest markers, Satt424 and AW132402, were used for genotyping *qCS8-2*. Although the CSIs of the RILs with YH alleles at *qCS8-2* were significantly higher than those of the RILs with TZ alleles in both years, the CSIs of the RILs with YH alleles at *qCS1-1* were significantly higher than those of the RILs with TZ alleles only in 2018, and not in 2019 (Fig. 6). As a result, we considered *qCS8-2* to be a stable QTL associated with SC tolerance.

### Validation of the effects of *qCS1-1* and *qCS8-2* on CSI

The SC tolerance of five RHLs for *qCS1-1* and *qCS8-2* was evaluated. The CSI of YT2-1-7 was significantly higher than those of TZ and YT2-1-28 (Table 2). The CSI of YT2-1-20-6 was similar to that of YH. The RHLs with TZ alleles at *qCS8-2* were SC-tolerant. The results of an ANOVA of the CSIs associated with each marker are shown in Table 3. At the Satt424 and AW132402 markers linked to *qCS8-2*, the CSIs of the TZ genotype were significantly lower than those of the YH genotype. Thus, the TZ genotype decreased the CSI value. The effect of *qCS8-2* was also validated using RHLs.

## Discussion

In two earlier studies, we showed that the *Ic* allele at the *I* locus contributes to SC tolerance in soybean (Senda *et al.* 2018, Yamaguchi *et al.* 2021). However, the *Ic* allele alone is insufficient to achieve higher SC tolerance (Yamaguchi *et al.* 2021). The aim of this study was to isolate another gene related to SC tolerance that can pyramided with the *Ic* allele to generate new, SC-tolerant cultivars. Therefore, the SC-tolerant cultivar TZ with the *II* genotype was analyzed in detail to determine its suitability as a new genetic resource. The characteristics of the seed coat of TZ under low temperature were analyzed to determine whether its SC-tolerance mechanism differs from that of T248. First, PA accumulation in the seed coat was surveyed by histochemical analysis using a DMACA staining method. After the 21-day low-temperature treatment, PA accumulation in the TZ seeds expanded to the dorsal side of the seed coat, similar to the pattern of PA accumulation in the SC-sensitive YH (Fig. 3), but different from that in the SC-tolerant T248 (Senda *et al.* 2018). These findings indicate that the SC tolerance mechanism of TZ is likely to differ from that of T248. Next, texture analyses were conducted to determine the physical properties of the seed coat. The results show that the hardness and flexibility of the seed coat differ between TZ and YH under low temperature, and that the physical properties of the TZ seed coat are not changed by low temperature (Fig. 4). In our prior study, only the breaking load was significantly decreased under low temperature in YH (Senda *et al.* 2018). This discrepancy may be caused by year-to-year differences. We previously detected two important physical properties of the SC-tolerant line T248 (Senda *et al.* 2018): (1) Although the low-temperature treatment did not decrease the breaking load of T248, the breaking loads of T248 were significantly lower than those of YH under both low- and normal-temperature treatments; and (2) compared with YH in the normal-temperature treatment, T248 in the low-temperature treatment showed significantly greater flexibility (breaking elongation and rupture time). In the present study, we detected physical properties of TZ that differ from those of T248. Specifically, we found that: (1) The breaking loads of TZ in the normal- and low-temperature treatments were not significantly lower than that of YH in the normal-temperature treatment (Fig. 4); and (2) the values of flexibility (breaking elongation and rupture time) were not significantly higher in TZ in the low-temperature treatment than in YH in the normal-temperature treatment (Fig. 4). These results suggest that the physical properties of the seed coat likely differ between the SC-tolerant cultivar TZ and the SC-tolerant line T248, providing further evidence that the SC-tolerance mechanism of TZ differs from that of T248. Comparisons between TZ and T248 at the same time and under the same experimental conditions are needed to verify this possibility. The results of histochemical and seed coat penetration analyses obtained in the present study indicate that the SC-tolerance mechanism of TZ differs from that of T248 with the *IcIc* genotype, and suggest that TZ harbors a novel SC tolerance-related gene.

QTL analysis for CSI was performed using a RIL population derived from a cross between YH and TZ. Two QTLs (*qCS1-1* and *qCS8-2*) associated with SC tolerance were detected (Table 1). However, *qCS1-1* was detected only in one year, and its effect on SC tolerance was not confirmed in RHLs (Tables 1–3). In contrast, *qCS8-2* was detected in both years, and its effect on SC-tolerance was validated in RHLs (Tables 1–3). These results indicate that only *qCS8-2* is a stable QTL associated with the SC tolerance of TZ. The physical properties of the TZ seed coat were not affected by low temperature (Fig. 4), probably due to the function of *qCS8-2*. Fine mapping and gene isolation may clarify the functions of *qCS8-2* in the future.

In our earlier study, we detected two QTLs associated with the SC tolerance of T248, *qCS8-1* and *qCS11-1*, using RILs derived from a cross between T248 and YH; we validated the effects of *qCS8-1* and *qCS11-1* on SC tolerance in RHLs (Yamaguchi *et al.* 2015). Additionally, we suggested that *qCS8-1* is likely the *Ic* allele (Senda *et al.* 2018, Yamaguchi *et al.* 2015). The *I* locus was mapped to a region on Gm08 (approximately 8.5 Mb) (Cho *et al.* 2019). In this study, the QTL *qCS8-2* was mapped to the genomic region between Satt424 and AW132402 (10.6 to 11.7 Mb) (Table 1). The physical distance between *Ic* and *qCS8-2* was estimated to be 2–3 Mb. Our results indicate that TZ possesses a gene for SC tolerance that differs from that in the SC-tolerant line T248. It will be possible to develop new cultivars with the *Ic* allele pyramided with the TZ allele at *qCS8-2*. We previously reported that gene pyramiding of a pubescence color gene (*T*) and the *Ic* allele can improve tolerance to SC (Yamaguchi *et al.* 2021). Further new cultivars with improved SC tolerance may be developed by pyramiding the TZ allele at *qCS8-2*, *T*, and the *Ic* allele. In conclusion, the findings of this study will contribute to stable soybean production in cold climates.

## Author Contribution Statement

NY and MS designed the research and wrote the manuscript. NY conducted the phytotron tests and QTL analysis. YS, FT, MI and MS conducted the DNA marker experiments. KY, MK and MS conducted the histochemical assays and penetration tests. All authors read and approved the manuscript.

## Supplementary Material

Supplemental Figure

Supplemental Table

## Figures and Tables

**Table 1.** Single marker analysis of cracked seed index (CSI) in the recombinant inbred soybean lines

Year	Chr*^a^*	Marker	Position (Glyma2.0)	Intercept (b0)	Slope (b1)*^b^*	Hypothesis test -2 ln(L0/L1)	*F*(1, n-2)	*P*	R^2^ (%)*^c^*	QTL name
2018	Gm01	Satt179	40222k	7.00	2.36	8.61	8.83	0.004**	8.0	*qCS1-1*
	Gm01	Satt436	49570k	6.84	1.65	4.36	4.37	0.039*	3.7	
	Gm08	Satt589	5182k	6.86	1.65	4.14	4.14	0.045*	2.4	
	Gm08	Sat_162*^d^*	8283k	6.85	1.83	5.12	5.15	0.026*	7.5	
	Gm08	Satt424	10633k	6.89	1.97	5.84	5.90	0.017*	10.5	*qCS8-2*
	Gm08	AW132402	11786k	6.93	1.81	4.88	4.90	0.029*	6.5	
2019	Gm08	Satt424	10633k	15.41	2.86	8.73	8.96	0.004**	12.0	
	Gm08	AW132402	11786k	15.47	3.07	10.11	10.45	0.002**	12.0	*qCS8-2*

*^a^* Chr, chromosome. *^b^* Effect of Yukihomare (YH) allele to increase CSI on the QTL. *^c^* Percentage phenotypic variance explained by the QTL. *^d^* Sat_162 is a marker proximal to the *I* locus (Oyoo *et al.* 2011). * Significant at *P* < 0.05. ** Significant at *P* < 0.01.

**Table 2.** Validation of the effect of *qCS1-1* and *qCS8-2* on cracked seed index (CSI) using five residual heterozygous lines in 2019

Cultivar or line	Generation	*qCS1-1* (Satt179)*^a^*	*qCS8-2* (Satt424, AW132402)	CSI*^b^*
Yukihomare (YH)		YH	YH	16.7 ab
Toyomizuki (TZ)		TZ	TZ	5.9 b
YT2-1-7	F_5_	YH	YH	22.5 a
YT2-1-28	F_5_	YH	TZ	5.6 b
YT2-1-25	F_5_	TZ	TZ	6.1 ab
YT2-1-20-6	F_6_	TZ	YH	17.7 ab
YT2-1-20-4	F_6_	TZ	TZ	9.0 ab

*^a^* YH, Yukihomare allele. TZ, Toyomizuki allele. *^b^* Within a column, means followed by the same letter are not significantly different according to Tukey’s HSD test (*P* ≥ 0.05).

**Table 3.** One-way ANOVA of cracked seed index (CSI) associated with each DNA marker using five residual heterozygous lines in 2019

Marker genotype	CSI	
	*qCS1-1* (Satt179)	*qCS8-2* (Satt424, AW132402)
Yukihomare (YH)	14.1	20.1
Toyomizuki (TZ)	10.9	6.9
Significance	ns*^a^*	***

*^a^* Not significant. *** Significant at *P* < 0.001.
