# Supplementary material for: A novel QTL associated with tolerance to cold-induced seed cracking in the soybean cultivar Toyomizuki
Source: Breed Sci. 2023 Apr 25;73(2):204–11. doi: 10.1270/jsbbs.22066 (PMC10316309; doi:10.1270/jsbbs.22066)
Supplement: Supplementary file 1 — Supplemental Figure [file 73_204_s1.pdf]

Yamaguchi et al./Breeding Science
doi: 10.1270/jsbbs.22066

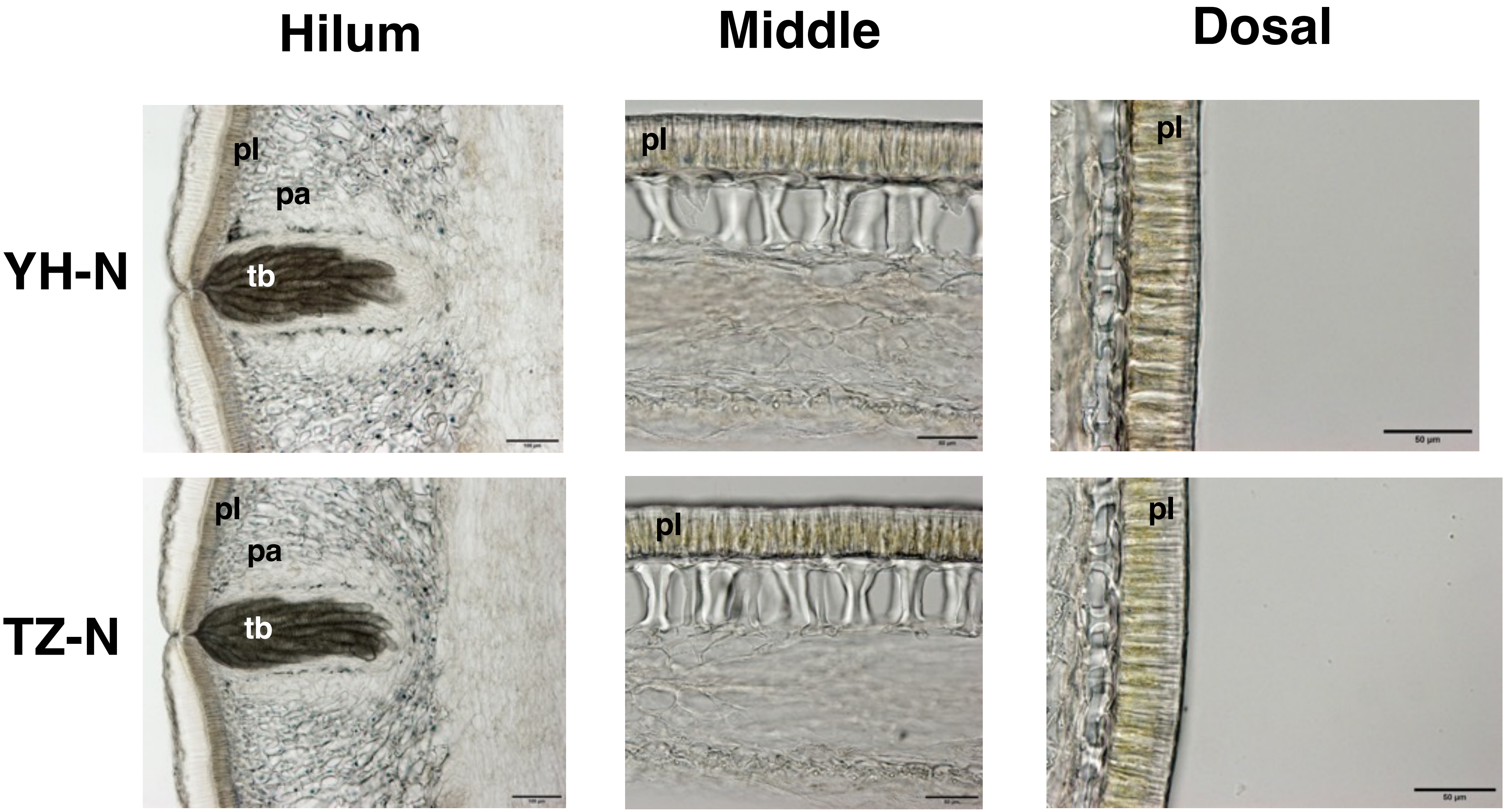

Supplemental Fig. 1. Cross sections of seed coats of Yukihomare (YH) and Toyomizuki (TZ) grown under normal conditions (YH-N and TZ-N, respectively). Seed coat sections were stained with DMACA to show proanthocyanidin accumulation. pa, parenchyma. pl, palisade layer. tb, tracheid bar. Scale bars are 100 µm (Hilum region) and 50 µm (Middle and dosal regions)
