# Supplementary material for: A novel QTL associated with tolerance to cold-induced seed cracking in the soybean cultivar Toyomizuki
Source: Breed Sci. 2023 Apr 25;73(2):204–11. doi: 10.1270/jsbbs.22066 (PMC10316309; doi:10.1270/jsbbs.22066)
Supplement: Supplementary file 2 — Supplemental Table [file 73_204_s2.pdf]

doi: 10.1270/jsbbs.22066

Supplemental Table 1. Single marker analysis of cracked seed index (CSI) in the recombinant inbred soybean lines using the 59 SSR markers

			Year 2018						Year 2019					
Chromosome	Marker	Position (Glyma2.0)	Intrecept (b0)	Slope (b1)	Hypothesis test -2 ln(L0/L1)	F(1, n-2)	P	R^2 (%)	Intrecept (b0)	Slope (b1)	Hypothesis test -2 ln(L0/L1)	F(1, n-2)	P	R^2 (%)
Gm01	Satt179	40222k	7.00	2.36	8.61	8.83	0.004**	8.0	15.44	0.95	0.92	0.90	0.345	1.4
Gm01	Satt436	49570k	6.84	1.65	4.36	4.37	0.039*	3.7	15.43	-0.38	0.15	0.15	0.699	0.4
Gm01	Gm01.2.2	50712k	6.82	1.26	2.68	2.66	0.106	2.3	15.44	-0.36	0.15	0.15	0.704	0.1
Gm01	Gm01.3.1	51819k	6.89	1.06	1.95	1.93	0.168	2.8	15.42	-0.23	0.06	0.06	0.807	0.1
Gm01	Sat_160	54121k	6.90	0.74	0.94	0.93	0.339	2.2	15.41	0.04	0.00	0.00	0.967	0.0
Gm01	Satt129	55166k	6.92	0.87	1.30	1.28	0.261	0.9	15.41	-0.10	0.01	0.01	0.918	0.1
Gm02	Sat_227	3124k	6.83	-1.32	2.17	2.15	0.146	2.2	15.52	1.65	2.32	2.30	0.133	2.6
Gm02	Satt600	29355k	6.87	-0.29	0.11	0.11	0.740	0.1	15.65	1.82	3.10	3.08	0.083	3.4
Gm02	Satt141	32004k	6.86	-0.30	0.12	0.12	0.735	0.1	15.65	1.59	2.34	2.32	0.132	2.2
Gm02	Sat_135	37285k	6.86	-0.30	0.12	0.12	0.735	0.1	15.65	1.59	2.34	2.31	0.132	2.1
Gm02	Sat_183	44317k	6.94	0.77	0.93	0.92	0.341	1.2	15.42	0.40	0.18	0.17	0.678	0.5
Gm02	Satt459	45311k	6.94	0.77	0.93	0.92	0.341	1.5	15.42	0.40	0.18	0.17	0.678	0.4
Gm02	Sat_198	45542k	6.93	0.96	1.47	1.45	0.232	4.3	15.41	0.26	0.07	0.07	0.790	0.5
Gm02	Sat_415	47039k	6.89	0.26	0.11	0.10	0.748	0.7	15.40	0.14	0.02	0.02	0.884	0.1
Gm03	Satt152	3366k	6.86	0.45	0.30	0.29	0.589	0.4	15.62	-1.93	3.90	3.90	0.051	4.4
Gm03	Satt009	3931k	6.87	0.27	0.11	0.11	0.747	0.4	15.63	-1.67	2.91	2.89	0.092	2.0
Gm03	Satt530	5669k	7.00	-0.91	1.25	1.23	0.270	0.4	15.55	-1.31	1.79	1.77	0.186	0.9
Gm03	Sat_208	20201k[a]	6.98	-0.71	0.75	0.74	0.392	0.5	15.52	-1.04	1.13	1.11	0.294	1.2
Gm05	Sat_356	3265k	6.91	0.04	0.00	0.00	0.964	0.0	15.30	-1.67	2.63	2.61	0.110	2.9
Gm06	Gm06.2.2	49287k	6.86	-0.36	0.20	0.20	0.659	0.3	15.26	-0.99	1.07	1.05	0.308	1.9
Gm06	Satt371	49759k	6.87	-0.34	0.18	0.18	0.672	0.3	15.25	-1.21	1.60	1.58	0.212	2.1
Gm07	Sat_391	1679k	6.90	0.28	0.12	0.12	0.734	0.1	15.40	1.45	2.26	2.24	0.138	1.0
Gm07	Satt150	2434k	6.79	0.88	1.23	1.21	0.274	1.4	15.21	1.57	2.74	2.72	0.102	1.6
Gm07	Satt540	5010k	6.60	1.19	2.07	2.05	0.156	6.1	15.00	1.67	2.85	2.83	0.096	4.2
Gm07	Satt435	5503k	6.64	1.16	1.99	1.97	0.164	4.7	15.01	1.79	3.32	3.31	0.072	4.2
Gm07	Satt536	12664k	6.88	0.36	0.20	0.19	0.662	0.0	15.36	0.86	0.78	0.77	0.383	0.9
Gm07	Satt175	15394k	6.91	0.37	0.20	0.20	0.657	0.0	15.44	0.91	0.86	0.85	0.359	1.5
Gm07	Satt680	21332k	6.93	0.94	1.32	1.30	0.258	0.6	15.45	1.18	1.41	1.39	0.241	1.6
Gm07	Satt697	33484k	6.93	0.94	1.32	1.30	0.258	0.6	15.45	1.18	1.41	1.39	0.241	1.6
Gm08	Satt589	5182k	6.86	1.65	4.14	4.14	0.045*	2.4	15.41	0.13	0.02	0.02	0.894	0.0
Gm08	Sat_162	8283k	6.85	1.83	5.12	5.15	0.026*	7.5	15.37	1.59	2.64	2.62	0.109	4.6
Gm08	Satt424	10633k	6.89	1.97	5.84	5.90	0.017*	10.5	15.41	2.86	8.73	8.96	0.004**	12.0
Gm08	AW132402	11786k	6.93	1.81	4.88	4.90	0.029*	6.5	15.47	3.07	10.11	10.45	0.002**	12.0
Gm09	Satt178	6912k	7.07	-1.00	1.41	1.39	0.241	1.7	15.24	1.18	1.36	1.34	0.249	0.8
Gm09	Satt349	7879k	6.97	-0.66	0.65	0.63	0.428	0.3	15.25	1.50	2.30	2.28	0.134	2.2
Gm09	Satt499	40583k	6.96	-0.97	1.49	1.47	0.228	0.7	15.42	-0.29	0.10	0.09	0.761	0.3
Gm09	Gm09.3.3	41460k	6.94	-0.80	1.01	0.99	0.322	0.5	15.42	-0.24	0.06	0.06	0.805	0.0
Gm09	Sat_167	43021k	6.86	-1.19	2.10	2.08	0.153	1.5	15.40	-0.35	0.12	0.12	0.729	0.2
Gm10	Sat_196	174k	6.73	-1.22	2.09	2.07	0.154	2.3	15.29	-0.95	0.88	0.86	0.356	1.7
Gm10	Gm10.1.1	3019k	6.83	-0.64	0.55	0.54	0.463	0.6	15.54	1.09	1.14	1.12	0.293	1.3
Gm10	Sat_038	46052k	6.93	-0.27	0.11	0.10	0.748	0.3	15.42	-0.12	0.01	0.01	0.909	0.0
Gm10	Satt243	46657k	6.93	-0.26	0.10	0.09	0.762	0.0	15.40	0.15	0.02	0.02	0.882	0.0
Gm13	CSSR535	24077k	6.91	0.02	0.00	0.00	0.986	0.0	15.40	-0.20	0.04	0.04	0.839	0.0
Gm13	Satt663	25936k	6.91	-0.03	0.00	0.00	0.972	0.1	15.41	0.02	0.00	0.00	0.986	0.1
Gm13	Gm13.1.3	41605k	7.00	-0.96	1.36	1.34	0.250	1.6	15.50	-0.88	0.79	0.77	0.381	1.0
Gm14	Sat_287	5287k	6.81	1.05	1.59	1.57	0.214	2.5	15.29	1.30	1.70	1.68	0.198	0.5
Gm14	Satt467	6480k	6.89	0.30	0.13	0.13	0.721	0.0	15.32	1.27	1.65	1.63	0.205	1.3
Gm15	Sat_124	11099k[a]	6.93	0.27	0.10	0.09	0.760	0.2	15.36	-0.97	0.88	0.86	0.355	1.3
Gm15	Satt685	49067k	7.12	-1.52	3.03	3.01	0.086	3.6	15.36	0.36	0.12	0.11	0.736	0.3
Gm17	GMES0252	471k	6.89	0.94	1.17	1.16	0.285	2.1	15.42	-0.37	0.13	0.12	0.728	0.1
Gm17	Sat_333	1746k	6.88	1.33	2.36	2.34	0.130	2.6	15.41	0.04	0.00	0.00	0.970	0.5
Gm18	Satt288	51127k	7.08	1.28	2.07	2.05	0.156	2.0	15.54	1.02	0.91	0.89	0.347	1.0
Gm19	Sat_408	395k	6.85	0.45	0.29	0.28	0.595	0.3	15.47	-0.42	0.18	0.17	0.679	0.2
Gm20	Satt419	1885k	6.90	-1.03	1.39	1.37	0.245	1.3	15.41	-0.43	0.16	0.16	0.690	0.2
Gm20	Satt292	40623k	6.86	0.89	1.34	1.32	0.254	2.8	15.45	-0.52	0.32	0.31	0.579	0.4
Gm20	Satt623	42165k	6.85	0.92	1.52	1.50	0.224	1.6	15.44	-0.36	0.16	0.15	0.696	0.2
Gm20	Sat_420	42934k	7.56	1.36	2.54	2.52	0.116	2.7	15.84	0.91	0.79	0.77	0.381	0.9
Gm20	Sct_189	46678k	7.06	0.82	0.99	0.97	0.327	1.6	15.45	0.19	0.04	0.04	0.852	0.0
Gm20	CSSR366	47620k	7.05	0.65	0.59	0.58	0.448	0.2	15.40	-0.06	0.00	0.00	0.956	0.3

[a] Position in Glyma1.0 version.
* Significant at $P < 0.05$.
** Significant at $P < 0.01$.
